# Food waste management practices in hospital foodservices and their associated greenhouse gas emissions: potential for increased environmental sustainability

**DOI:** 10.3389/fnut.2025.1541657

**Published:** 2025-05-13

**Authors:** Yee Man Janis Yip, Nathan Cook, Jorja Collins

**Affiliations:** Department of Nutrition, Dietetics and Food, Monash University, Notting Hill, VIC, Australia

**Keywords:** emissions, environment, foodservices, food waste, greenhouse gas, hospital, sustainability, waste management

## Abstract

**Introduction:**

Hospitals produce and waste large amounts of food. When disposed in landfill it creates greenhouse gases (GHGs) from the decomposition process. While various food waste management strategies exist that divert hospital food waste to an alternative end of life pathway to landfill, it is not clear which can decrease GHG emissions the most. This study aimed to (a) compare the differences in GHG emissions associated with hospital foodservice food waste before and after adopting a food waste management strategy, and (b) identify which waste management strategy can prevent the most GHGs in 1 year.

**Materials and methods:**

A secondary analysis of data from a systematic review reporting on food and food-related waste diversion strategies in hospital foodservice was conducted. The online “ReFED Impact Calculator” was used to calculate GHG emissions from food waste in the original scenario (e.g., landfill), and the alternative scenario after a food waste management strategy that reused, recycled or recovered resources was implemented. The net change of GHGs was calculated, and the GHGs emissions avoided in paired samples and between food waste management scenarios was analyzed statistically.

**Results:**

Fifty-five food waste management strategies (surplus food donation, feeding animals, anaerobic digestion or industrial uses, and composting) were eligible for analysis and were grouped into eight scenarios. The median GHGs generated decreased after adopting the alternative strategy in all scenarios. There was a statistically significant median reduction in GHGs when changing from landfill to donations (−11.54, *p* < 0.001), landfill to industrial uses (−25.92, *p* < 0.001), and landfill to composting (−15.24, *p* < 0.001). Percentage change in GHGs generated in these 3 scenarios demonstrated a significant difference (*p* < 0.001), with landfill to donations displaying the greatest reduction in GHGs (−92.02%), followed by composting (−8.69%) and industrial uses (−7.75%).

**Conclusion:**

Various food waste diversion strategies can handle types and volumes of hospital food waste, yet each strategy displays a reduction in GHG emissions compared to a lower prioritized strategy. Donating waste shows the greatest reduction in GHG emissions and if food waste cannot be avoided, it may be the preferred end of life pathway for food waste.

## 1 Introduction

Climate change is a threatening environmental problem affecting the globe. This is largely caused by intensive industrial activities and urbanization in different countries, increasing the level of carbon emissions to the atmosphere and concomitantly increasing the generation of greenhouse gasses (GHGs), including carbon dioxide (CO_2_) and methane (CH_4_), subsequently causing a global rise in the planet’s temperature and weather extremities (1, 2). Climate change results in ecosystem degradation, such as natural resources depletion and pollution, can lead to wildlife extinction, jeopardize human health through diseases and food insecurity (1, 2). In 2018, there was a 2% increase in global GHG emissions and CO_2_ was found to be the primary contributor to overall emissions (2, 3). In 2019, CO_2_ emissions had an increase of 0.34% (122 million tons) over the past year (2). There is an urge to address causes of climate change as it is foreseeable that global temperature will rise 1.5°C, between 2030 and 2052 if no actions are taken to mitigate GHG emissions (2–4).

Waste disposal in landfills is one of the crucial factors contributing to the increased atmospheric GHGs concentration, as soil microbes decompose the waste and generate CO_2_ and CH_4_ as byproducts (2, 5). Methane (CH_4_) is a strong GHG that has a 25-times greater global warming potential compared to CO_2_ (6, 7). Food waste is identified as a prime waste stream to address due to its organic nature and being the greatest contributor to CH_4_ emissions (4). Food is often decomposed or wasted throughout the food supply chain (FSC) (i.e., from initial agricultural stage to final consumption stage), which has a direct impact on GHG emissions and the environment (8). Food waste is defined as “*edible material that arises throughout the FSC which originally intended for human consumption but is instead thrown away, lost or degraded, damaged by pests, or used for other purposes such as animal feeding or industrial use that inclusive of inedible parts*” (8), p.108. Globally, 1.3 billion tonnes of food is wasted or lost annually, in which 22% of the food waste comes from the consumption stage of the FSC (9, 10), inclusive of households, retail/commercial food businesses, institutions (11). Food wastage costs the global economy nearly USD 940 billion and produces 4.4 gigatonnes of CO_2_ equivalents (CO_2_e) annually, contributing to 87% of global warming emissions (12). To tackle the global food waste problem, the United Nations has developed the Sustainable Development Goals (SDGs) including SDG 12.3 of “halving per capita food waste globally at retail and consumer levels and reduce food losses along the food production and supply chains by 2030” (13). Different national and local governments have also established policies in response to this (8, 14, 15), including the Waste (England and Wales) Regulations 2011 that were framed according to the European Union Waste Framework Directive (14), and the Australia National Food Waste Strategy (15), developed to reduce food waste.

Environmental impact from the healthcare sector is gradually being recognized due to its role of foodservice provision to patients, staff and visitors, creating enormous potential for food waste generation and management across the hospital FSC (i.e., from procurement to waste disposal) (4). If the global emissions from healthcare was a country, it would be the fifth largest contributor to global warming (16). In 2013, healthcare accounted for 9.8% of GHG emissions in the US, where at least 20–30% of total hospital waste was food waste, which can reach up to more than 50% in some facilities (4, 17). Food waste can originate from multiple sources within a hospital setting, including foodservice for patients and onsite retail/commercial food businesses for patients, staff and visitors (e.g., cafeterias, canteens, food courts, restaurants, kiosks, vending machines) (4). Factors contributing to high food waste rates in healthcare facilities were suggested in multiple studies investigating the patient end of the hospital food system, including foodservice models used, patients’ satisfaction regarding the food quality and quantity, and the clinical condition of patients (4, 17, 18). To elaborate, extensive time lag between ordering and delivery of meals, a pre-plated meal model, fixed mealtimes, unappetizing food, therapeutic diet prescriptions, and mealtime disturbances can all reduce patients’ interest in food consumption and thus contributing to food waste in hospitals (17–19).

Due to the responsibility of hospitals to provide ample chances for patients to achieve nutritional adequacy for recovery and maintain quality of life through their foodservice provision, it seems unavoidable for hospitals to generate food waste (4, 11, 18). Measurement and routine monitoring of food waste at a hospital level can be considerably challenging for healthcare operators. Recent research examining aggregate food waste audits show they require significant labor (staff, students or contractors), time (commonly 2 weeks) and equipment (scales, containers, protective personal equipment etc.), which are perceived by foodservice staff as a barrier (20, 21). Staff resistance to change, lack of organizational support, limited staff buy in and skill gaps and absence of policy enforcing waste measurement were also identified as challenges (21). Routine food waste measurement is notably important to benchmark and acknowledge opportunities for waste reduction and monitor progress, giving potential for financial savings and environmental improvements (4, 6, 11, 19, 20). Inability to efficiently or reliably measure and monitor food waste limits action on food waste. The use of digital technology and artificial intelligence offers promise for food waste measurement, although greater adoption in healthcare is needed (22). Bux propose a method using intelligent 3D cameras, visual computing, machine learning and algorithms to generate qualitative and quantitative food waste data within a dashboard that speeds up the process, allows for real time waste tracking, monitors trends over time, conveys economic, environmental and social implications of waste and provides feedback on patients’ nutritional intake (22).

The traditional end of life pathway for food waste in hospitals is landfill. Implementing a food waste management strategy with an alternative end of life destination can improve the environmental and socio economic sustainability of their sites (4, 11). The food recovery hierarchy (23) was introduced and developed based on the waste hierarchy framework, highlighting how food waste can be a resource, and that the appropriate management of wasted food, according to the framework, can confer the best outcomes to the environment, economy, and society (8, 23). The hierarchy ranges the food waste management strategy options from the most (top) to least (bottom) favorable in regard to the utilization of that food waste (23). Minimizing food waste in the first place is the most preferable option (23), yet this can be challenging to achieve in complex settings such as healthcare facilities (4, 11). For unavoidable food waste, the hierarchy recommends reusing the food surplus by donating to populations in need, followed by diverting the food waste to animals as feed, resource recovery using industrial processes such as anaerobic digestion (AD) or other methods, then composting for soil nourishment (11, 23). Disposing food waste to landfill, incineration or sewer is the least favorable strategy and last on the hierarchy (15, 23).

A systematic review focused on the environmental outcomes of hospital foodservices across the FSC found only 8 peer-reviewed studies reporting on waste management practices that divert food waste from landfill (4). Cook et al. extended this research to include gray literature in a systematic review to understand the scope of food waste management practices in hospitals worldwide (11). This review found a total of 85 records using strategies to divert food and food-related waste (i.e., packaging materials) from landfill across hospital settings (11). Different measurements were used in the included records to describe the environmental outcomes, such as quantities of food or food-related waste in weight or number of meals diverted, avoidance in CO_2_e emissions, water savings, and biogas and/or energy generation (4, 11, 18). The non-uniformity in quantifying outcomes can be difficult to determine which food waste management strategy gives the greatest reduction in GHG emissions and contributes to environment sustainability in hospitals. Additionally, the lack of baseline data or control group is a limitation in the included studies to make robust conclusions of the beneficial outcomes of various food waste diversion approaches (4, 18). It is essential to determine promising waste management strategies that can divert food waste from landfills in healthcare foodservices to mitigate GHG emissions and climate change. To address the gap in the literature and enlighten institutions to manage food waste sustainably, this study aims to (a) compare the differences in GHG emissions from hospital food waste before and after a food waste management strategy was adopted, and (b) identify which waste management strategy from the food recovery hierarchy (23) can prevent the most GHGs in 1 year. GHG emissions from food waste included emissions associated with activities across the FSC, not just waste disposal.

## 2 Materials and methods

### 2.1 Study design

This study is a secondary analysis of data from a published systematic review that was completed and reported according to the Preferred Reporting Items for Systematic Reviews and Meta-Analyses (PRISMA) guidelines and aimed to describe the types, characteristics, outcomes and barriers and enablers of food and food-related waste management strategies in hospital foodservice. A thorough search of six databases and gray literature was completed, followed by a two step selection processes completed in duplicate. Further details on the aims, methods and findings of the review can be found in the full open access article (11). The dataset from the original review formed the basis of this quantitative study. In summary, this secondary analysis involved inputting food waste data from the systematic review into a web-based calculator to determine the GHG footprint of current/usual end of life pathway and an alternative. An excel spreadsheet and statistical software package were used to manage, report and analyze data within and across scenarios of current/usual and alternative food waste management strategies. The original review and this secondary analysis did not involve recruitment of human subjects, so ethics approval was not required.

### 2.2 Sample

The dataset generated in the original review comprised of 85 records which included 4 peer-reviewed and 81 gray literature documents that reported waste management strategies used in hospital foodservices internationally to manage food and food-related waste (11). The data included strategies that reused, recycled or recovered resources from these wastes as proposed by the food recovery hierarchy, including surplus food donations, feeding animals, AD or industrial uses, and composting (8, 23). Strategies related to food waste prevention were not included since the review was focused on waste management strategies (11).

The review compiled results on financial, environmental, or staffing outcomes after implementation of an alternative end of life pathway (11). The amount of food waste diverted from the standard/current destination to a more preferable destination was one of the environmental outcomes, and this data was relevant for this secondary analysis. The authors analyzed the 85 records to determine records which were eligible to include in the secondary analysis reported here. The eligibility criteria are outlined in Table 1. All records that reported a food waste amount in weight diverted from a lower prioritized waste management strategy to a higher prioritized strategy on the food recovery hierarchy (23) (e.g., composting to donation) in a specified time-period were included in this secondary analysis. Records using number of meals to quantify the food waste, records without units for the amount of food waste, or records not reporting a time-period for food waste diversion were excluded as data could not be analyzed reliably. Food waste consisting of a single source (e.g., coffee grounds or oil) were excluded as the GHG emissions profile would be different to those from a ‘standard mix’ of food.

**TABLE 1:** Eligibility criteria for studies included in the analysis of GHGs avoided under food waste management strategies in hospital foodservices.

Inclusion criteria	
**Parameter**	**Description**
Population	Hospital foodservice that provides food for patients, onsite retail/commercial foodservice to patients, staff and visitors at the hospital (e.g., canteens, cafeterias, restaurants) and offsite central production kitchen (CPK) that produces food for hospitals
Intervention	An alternative end of life pathway for food waste (i.e., alternative food waste management strategy) that is higher on the food waste recovery hierarchy (23) than the comparator.
Comparator	The usual end of life pathway for food waste (i.e., standard food waste management strategy) (e.g., landfill, incineration, sewer)
Outcome	The weight of food waste reported in the intervention scenario compared to the comparator scenario in a specified time frame, used by the authors to calculate the difference in GHG emissions between the two strategies.

### 2.3 Data collection and outcomes

Researchers developed a template in Excel (Version 2022, Microsoft Corporation, Washington) to extract, manage and analyze data from included records (11). Collection fields included citation (author and year), location of facility, number of facilities, setting type, food waste source, current food waste management strategy (before), alternative food waste management strategy (after), weight of food waste, GHG footprint under current and alternative strategies, and the difference in GHG footprint (calculated using the custom excel spreadsheet). We grouped the data together into different scenarios based on the current/before and the alternative/after destination for food waste.

If the current food waste management practice was not explicitly reported, landfill was assumed to be the current strategy. This assumption was made because landfill was a common practice in global healthcare foodservices to manage food waste (6, 11, 24–26). For consistency of units and timeline of food waste diversion for comparison, all units of weight were converted to kilos by using the conversion of 1 ton = 907.185 kilos. For cases that had reported an approximate or a range of diverted food waste quantity, the estimated or maximum amount was used for calculations. For cases that included multiple facilities, an average of food waste weight was calculated to estimate food waste diverted in one facility. Depending on the reported timeline of diversion, the waste amount was then multiplied or divided to estimate the total weight of food waste diverted from landfill in a 1-year period.

GHG footprint is the total GHG emissions directly associated with the production of a product, including the upstream stages in the FSC to end-of-life (27). It is often measured in metric tons of carbon dioxide equivalents (MTCO_2_e), and to convert a GHG emissions into CO_2_e, its emissions are multiplied by the gas’s global warming potential (GWP), as it allows for difference in effectiveness of warming the Earth when compared to CO_2_ per unit mass (28). The outcomes of interest in the current study were GHG footprint in MTCO_2_e generated from food waste under current and alternative food waste management strategy respectively, and net change of GHG footprint in MTCO_2_e. This was achieved by calculating and comparing the GHG emissions saved in a 1-year period under various food waste management strategies within a hospital foodservice setting.

The online Rethink Food Waste Through Economics and Data (ReFED) impact calculator^1^ was used to generate outcome data. The online calculator analyses data entered by the user about the sector, type and amount of food waste and how it is being disposed of to calculate the GHG footprint. It allows data for an alternative scenario to be inputted, and compares scenarios to give the net benefit or the “impact” (29). It utilizes GHG emissions data generated by Quantis sustainability consultancy firm through a life cycle assessment approach (30). ReFED is a reputable non-profit organization in the US that aims to reduce food waste across the food system by formulating solutions using evidence-based data to achieve environmental and socioeconomic sustainability (29). The impact calculator was chosen for use in this study compared to other available models because it allows the direct comparison of environmental impacts from changes in food waste management practice, aligning with the research aims. The calculation of total GHG footprint by the ReFED calculator accounts for emissions throughout the FSC from production, to food waste disposal. Total GHG footprint is calculated as upstream GHG emissions (emissions accrued to produce, store and transport food up to the sector selected) + downstream GHG emissions (emissions associated with food disposal or redistribution) (31). Additionally, the online calculator is free of charge and easy to use for analysis.

Food waste weight data were input to the calculator to generate the GHG emissions associated with food waste under current and alternative scenarios. The results from the calculator were entered into the custom excel spreadsheet designed for this analysis by the first author and reviewed by the research team. Before inputting the weight of food waste into the calculator, the following options were chosen to ensure consistency of data outcomes: “foodservice” as sector; “standard mix” as food type; “kg” as unit. “Standard mix” was chosen as most case studies reported a combination of food waste and some studies did not report on the food waste components. ReFED justified the option of “standard mix” as one of the most usual cases in food businesses where their waste source was numerous or unspecified (32). In the calculator, AD was listed as an individual food waste destination, which differs from the food recovery hierarchy that included AD under industrial uses (23). Therefore, for cases that mentioned using AD to divert food waste, this option was chosen as the alternative scenario on the calculator. For studies that reported using other techniques, such as greywater digester, biodigester, and dehydrator, to divert the food waste, industrial uses was selected as the alternative scenario as it was assumed the food waste was repurposed into industrial biomaterials (33). After inputting the quantity of food waste under current and alternative scenarios onto the calculator, the GHG footprint in each situation, together with the amount of GHG savings (net benefit) were calculated. The researcher team decided to express the net benefit as a net change of GHG emissions, which was calculated by subtracting the GHG emissions in the alternative strategy from the current strategy using the custom excel spreadsheet.

### 2.4 Data analysis

Statistical Package for Social Sciences (SPSS) (Version 28, SPSS INC; Chicago, IL, USA) was used to quantitatively analyze the data. Descriptive statistics were used to present the GHGs generated and avoided under different food waste management strategies. The percent change in GHG emissions were calculated to accommodate the variance of food waste quantities across facilities and the unequal sample sizes under different scenarios. Statistical significance for comparing GHG emissions between current and alternative strategies were paired in samples and calculated using sign test, while percentage change in GHGs between food waste management scenarios were compared using Kruskal-Wallis test, as data were non-parametric, and the distributions of differences between the paired samples and different scenarios were asymmetrical. Statistical significance was set at a value of *p* = 0.05.

## 3 Results

### 3.1 Sampling

**FIGURE 1:**
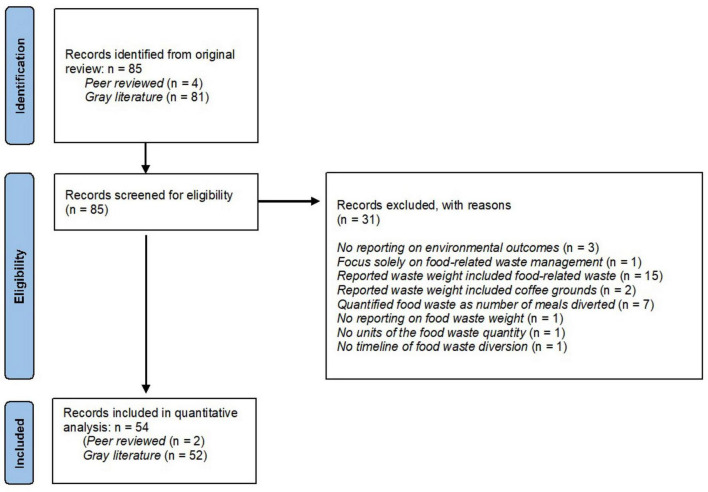
Flow diagram of included records for quantitative analysis of GHG emissions using food waste management strategies in hospital foodservices.

A total of 85 records which reported 85 food and food-related waste management strategies were identified in the original review and screened for eligibility (11). Thirty-one records were excluded due to various reasons indicated in Figure 1. Three cases reported on coffee grounds (*n* = 2) or cooking oil (*n* = 1) being recycled, yet the quantity of those were separated from the food waste weight reported and thus, they were considered eligible cases (34–36). A total of 54 records met eligibility criteria and were included in this study, in which 2 were published research studies and the remaining were gray literature documents. One hospital reported using two different strategies to manage food waste and therefore yielded 55 food waste management strategies used in the 54 records. Nine cases also reported on the quantity of avoided CO_2_e from food waste diversion (37–44). As the net savings of CO_2_e for each case was calculated from the ReFED impact calculator in the current study, the amount of avoided CO_2_e reported in those records were not used to allow for uniform comparison.

### 3.2 Hospital characteristics

Majority of facilities were in the US (*n* = 25), followed by Australia (*n* = 13), UK (*n* = 6), Canada (*n* = 5), Italy (*n* = 2), Brazil (*n* = 2), Spain (n = 1), and Singapore (*n* = 1). Most cases were taken place in hospitals (*n* = 37), whereas the remaining settings were in medical centers (*n* = 11), health services (*n* = 5), and central production kitchens (CPK) (*n* = 2). Sixty-seven percent (*n* = 37) of the sites diverted food waste from patients foodservice, while 13% (*n* = 7) diverted food waste solely from retail/commercial foodservices for patients, staff and visitors including cafeterias and canteens. Twenty percent (*n* = 11) of the sites diverted a combination of patients and retail/commercial foodservices food waste.

**FIGURE 2:**
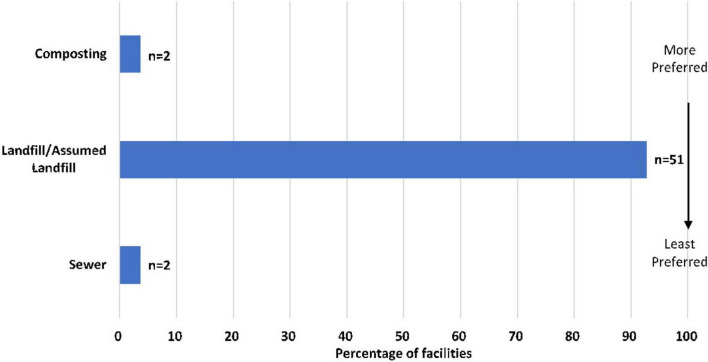
Current waste management strategy to manage food waste across facilities according to position in the food recovery hierarchy (23) (*n* = 55).

**FIGURE 3:**
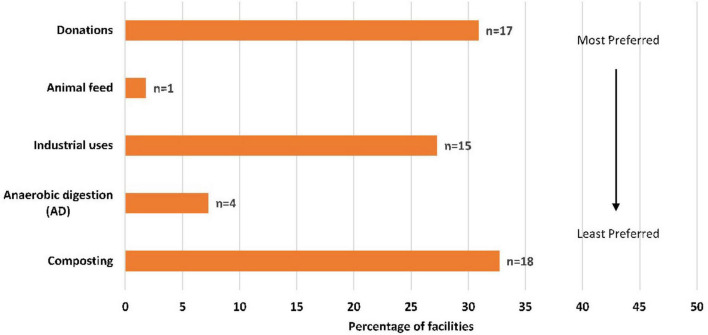
Alternative waste management strategy to manage food waste across facilities according to position in the food recovery hierarchy (23) (*n* = 55).

Figures 2, 3 depict the current and alternative food waste management strategies used respectively across the facilities. Amongst the current strategies, 93% of sites directly (*n* = 29) or were assumed (*n* = 22) to send waste to landfill, followed by sewer and composting which accounted for 4% (*n* = 2) each, respectively. Regarding the alternative strategies, 33% of sites employed composting, which was the most common waste management practice. Surplus food donations and industrial uses were also popular strategies to use, accounting for 31 and 27%, respectively. The remaining strategies included AD (7%) and feeding to animals (2%). Hospitals in the US appeared to be in favor of adopting food donations as an alternative strategy (*n* = 11). Only one hospital in Australia diverted food waste to animal feed. Industrial uses and composting were employed across several hospital locations, whereas AD was less common and practiced only in US and UK facilities.

Detailed characteristics on the setting and current and alternative strategies for food waste management are reported in Supplementary Table 1 (45–81).

### 3.3 Outcomes

**FIGURE 4:**
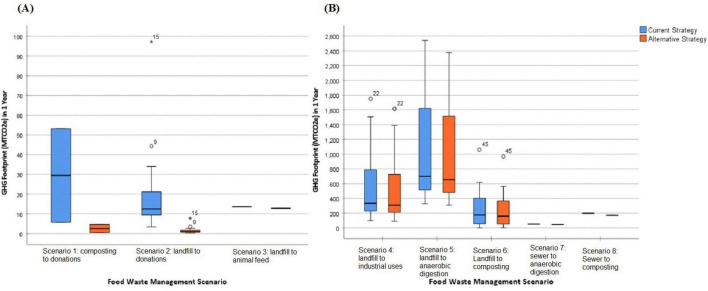
Boxplot graphs displaying median of GHG emissions (MTCO_2_e) across hospital foodservices in a 1-year period under eight food waste management scenarios (current strategy versus alternative strategy). **(A)** Displays Scenario 1 = Composting to Donations; Scenario 2 = Landfill to Donations^∧^; Scenario 3 = Landfill to Animal feed. **(B)** Displays Scenario 4 = Landfill to Industrial uses^∧^; Scenario 5 = Landfill to Anaerobic digestion (AD); Scenario 6 = Landfill to Composting^∧^; Scenario 7 = Sewer to Anaerobic digestion (AD); Scenario 8 = Sewer to Composting. ^∧^Indicates a statistically significant median net reduction in GHGs when the alternative strategy is adopted. *Indicates a case that is an extreme outlier.

There were eight food waste management scenarios based on the current/before and the alternative/after end of life pathway for food waste (Table 2). The most common scenarios were landfill to composting (*n* = 17); landfill to donations (*n* = 15); landfill to industrial uses (*n* = 15). Figure 4 shows the median GHG emissions under current and alternative strategy across the scenarios. The average GHGs generation decreased across facilities after adopting the alternative strategy to manage food waste in all scenarios. An exact sign test was conducted to compare the median GHGs of current strategies with the median GHGs of alternative strategies in paired samples under each scenario (Table 2). There was a statistically significant median net reduction in GHGs when facilities changed from landfill to donations (−11.54, *p* < 0.001), landfill to industrial uses (−25.92, *p* < 0.001), and landfill to composting (−15.24, *p* < 0.001).

**TABLE 2:** Descriptive statistics of GHG footprint (MTCO2e) generated and avoided in a 1-year period across different management scenarios.

Scenario	Current strategy	Alternative strategy	Median GHG –current strategy (IQR)	Median GHG –alternative strategy (IQR)	Median GHG – net change^#^ (IQR)	Significance* (*p*-value)	Minimum GHG net change^#^	Maximum GHG net change^#^	Median % net change^#^ in GHG
1	Composting	Donations (*n* = 2)	29.52	2.58	−26.95	0.500	−5.28	−48.61	−91.31%
2	Landfill	Donations (*n* = 15)	12.54 (14.33)	1.00 (1.14)	−11.54 (13.19)	<0.001	−3.07	−89.47	−92.02%
3	Landfill	Animal feed (*n* = 1)	13.61	12.81	−0.80	N/A	−0.80	−0.80	−5.88%
4	Landfill	Industrial uses (*n* = 15)	334.36 (630.67)	308.44 (581.78)	−25.92 (48.89)	<0.001	−7.54	−135.64	−7.75%
5	Landfill	Anaerobic digestion (*n* = 3)	699.97	653.85	−46.12	0.250	−21.69	−167.52	−6.59%
6	Landfill	Composting (*n* = 17)	175.38 (364.58)	160.14 (332.89)	−15.24 (31.69)	<0.001	−0.06	−92.10	−8.69%
7	Sewer	Anaerobic digestion (*n* = 1)	52.67	46.52	−6.15	N/A	−6.15	−6.15	−11.68%
8	Sewer	Composting (*n* = 1)	197.90	170.88	−27.02	N/A	−27.02	−27.02	−13.65%

^#^Expressed as net amount of GHG savings from current strategy (i.e., net change in GHG = alternative strategy GHG – current strategy GHG).

*Comparison of GHG emissions between current and alternative strategies in paired samples within scenario. *P*-values were calculated with the exact sign test.

**FIGURE 5:**
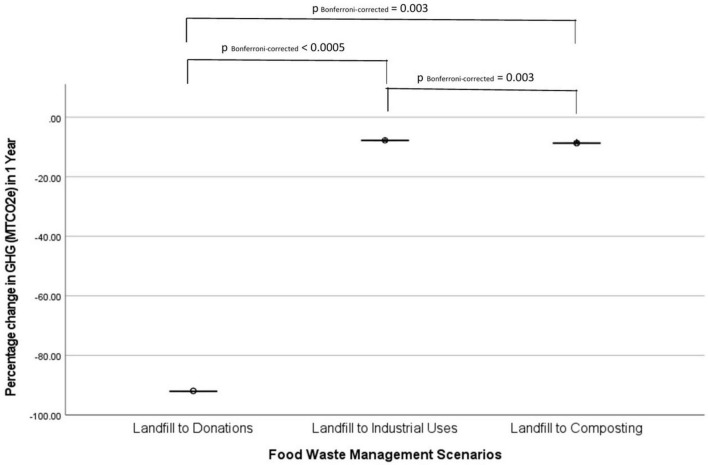
Pairwise comparisons on percentage change in GHG (MTCO_2_e) across three pairs of food waste management scenarios in hospital foodservices in a 1-year period using Kruskal-Wallis test with a *post-hoc* Bonferroni correction for significance values (*p*-values).

Regarding the percentage change in GHG emissions, all scenarios displayed a negative median percentage change (Table 2). A Kruskal-Wallis test was conducted to compare the median percentage change in GHG emissions between scenarios. Five scenarios were omitted from the analysis due to limited sample size (*n* ≤ 5) in those groups (82). The remaining 3 scenarios, including landfill to donations, landfill to industrial uses, and landfill to composting, were included in the analysis. The median percentage change in GHGs were significantly different between the scenarios, χ2(3) = 40.86, *p* < 0.001. Pairwise comparisons were performed using Dunn’s procedure with a Bonferroni correction for the pairs of scenarios. There was a significant difference between the 3 pairs of groups, with landfill to donations displaying the greatest reduction in GHGs (−92.02%), followed by landfill to composting (−8.69%) and landfill to industrial uses (−7.75%) (Figure 5).

## 4 Discussion

This study aimed to (a) compare the differences in GHG emissions from a hospital foodservice before and after a food waste management strategy was adopted, and (b) identify which waste management strategy to divert food waste from landfill according to the food recovery hierarchy (23) can prevent the most GHGs in 1 year. Fifty-five food waste diversion strategies were identified, which included healthcare facilities that changed their food waste management strategy to a more preferable food waste management strategy according to the food recovery hierarchy (23). All facilities achieved a greater reduction of GHG emissions when compared to their previous strategy. However, different food waste management scenarios have various potentials in avoiding GHGs. The scenarios in this study are comparable to some extent with the priority tiers in the food recovery hierarchy (23), whereby diverting food waste from landfill to donations displayed the greatest reduction in GHGs and is the most preferred waste management strategy. However, composting has the next greatest potential to reduce GHG emission but it is lower down the waste management hierarchy (23).

In this study, a large variability in average GHG emissions and savings is observed across the scenarios (Table 2), which is directly related to the amount of food waste that each scenario can manage. For scenarios where the current strategy is landfill, the analysis demonstrated the least amount of GHG emissions when using donations as an alternative strategy. This is also reflected in the median percentage change in GHG emissions, showing that food donations exhibit the greatest GHGs reduction (−92.02%), as it is assumed the donated food is consumed and a negligible amount of GHGs is emitted (32, 33). However, the amount of unserved and edible excess food in hospital foodservices that can be reused to feed people via food banks or food rescue can be limited (16). Reusing food from hospitals has also been suggested to be challenging due to contamination risks (18). In addition, legislations and guidelines, liability concerns about donated food causing illness, absence of onsite cooking facilities, and use of a menu cycle can be barriers restricting the reusability of surplus food within hospital settings due to higher risks of microbial contamination and food hygiene constraints (11, 18, 83). Even though donating food waste displays the highest GHG emissions reduction when compared to landfill, the small capacity of surplus food in hospital foodservices that can be donated implies a combination of food waste diversion strategies may be required to reduce GHG emissions and improve the environmental sustainability of the hospital foodservice.

In comparison, animal feed, industrial uses, AD and composting strategies are capable to manage a larger amount of food waste, including excessive food and food scraps produced during meal preparation, and plate waste that has already been served but not consumed (17, 23). This is demonstrated in the current study, where a larger amount of GHG emissions is seen when these strategies are used, with AD having the greatest GHG emissions across all alternative strategies. This may be due to the larger variety of food waste (e.g., fats and oils, food scraps, sewage sediment) that can be anaerobically digested into renewable energy and fertilizer (23, 83). In this study, composting displayed the second greatest reduction in GHG emissions from landfill (−8.69%), followed by industrial uses (−7.75%), AD (−6.59%) and animal feed (−5.88%). This is surprising as composting is situated in a lower priority tier in the food recovery hierarchy (23), but has the capacity for larger GHG emission savings when compared to other strategies higher up the hierarchy, excluding donation. This may be due to the large variance in sample size across scenarios, variability in methods used to collect and measure food waste data, and different composition in food waste across facilities (25, 84). It should be acknowledged that all alternative strategies display a reduction in GHG emissions compared to less preferred strategies (e.g., landfill), particularly in the 3 scenarios that divert food waste from landfill, showing significant decreases in GHG emissions (donations (−11.54, *p* < 0.001), industrial uses (−25.92, *p* < 0.001), and composting (−15.24, *p* < 0.001). Thus, adopting feasible waste diversion strategies to manage food surplus and the remaining food waste instead of sending this food waste to landfill or other less favorable approaches in hospital foodservices is essential for environmental outcomes (4, 11).

Composting, food surplus donations, and industrial uses are deemed to be the top 3 most common strategies used in hospital foodservices from the sample used in this study. Composting is often used despite the fact that it is a lower prioritized strategy in the food recovery hierarchy (23), which may be due to the lower costs and fewer operational issues compared to industrial methods in managing food waste effectively (11). Surplus food donations are popular practices in some countries (e.g., US), which may be due to high demand for food relief, established processes and legal protections for donating food (6, 85). Some countries (e.g., Sweden) also advocate the practice of food waste diversion by legislating bans on food waste to landfill or sending compostable waste into landfills (18, 25). Anaerobic digestion might be less often used in hospitals because of the higher financial costs compared to gains from the output products (11). Additionally, animal feeding appears to be the least popular strategy in hospital foodservices, and could be due to regulations that govern the type of food waste suitable for animal feed (25, 83, 86). According to the US Environmental Protection Agency food waste measurement report, the food manufacturing sector contributes a majority of their food waste (91%) to feed animals (86). This is because food waste appropriate for animal feed should be handled properly to ensure homogeneousness and absence of non-food substances (e.g., packaging materials) (86). Further, insufficient available resources to segregate mixed-types of food waste in a busy hospital food service setting, which may be a costly and difficult practice (16) could deter this practice, as the appropriate food cannot be separated.

Although not investigated in this study, strategies used to prevent food waste in the first place is vital for environmental and socioeconomic sustainability of the food system locally and globally (23, 83). This is also recommended as the most favorable strategy in the food recovery hierarchy (23). Several food waste studies point out that food waste occurring further along the FSC contains a higher carbon footprint than previous stages because of the resources used in subsequent stages in the FSC, indicating the GHGs impact aggregates to the highest level at the stage of food waste disposal (8, 12, 18). Reducing food waste can therefore bring direct environmental benefits by minimizing GHG emissions as well as reducing resource use along the FSC (83). This aids the conservation of land, water and other natural resources for feeding the global expanding population and alleviating food insecurity (8, 83). In addition, various food waste management options in the food recovery hierarchy, except source reduction, (23) could have different extents of environmental impacts (25, 83). Landfill or incineration has the biggest impact to GHG emissions without any resource recovery contributing back to the environment (4, 83). To a lesser extent, GHGs could be produced from the windrows production and machinery usage during composting (25). There is also considerable waste disposal costs associated with transportation and operations for each waste management strategy to consider (8, 11, 83).

Literature focusing on sustainable food waste management in hospital foodservices provides evidence-based solutions to reduce food waste and increase patient satisfaction (4, 17–19, 87). Some effective approaches include a flexible foodservice and meal delivery system that allow patients’ customization on food choices and portions, reducing time lag between meal ordering and delivery (e.g., room service model, bulk trolley meal service), and providing social opportunities to interact with foodservice personnel (e.g., spoken menu ordering) (4, 17–19, 87). Not to mention, upstream stages of hospital FSC (i.e., from procurement to production), are also pivotal in reducing food waste generation, minimizing GHG emissions and financial costs (6). Practical strategies include prioritizing the use of locally-grown, organic and seasonal food produce, sourcing sustainable food and food-related products (e.g., biodegradable packaging), offering more plant-based proteins to replace animal proteins in menus, use of an electronic menu and meal forecast system, and accurate food portioning during service (6, 8, 88). Each of these aforementioned approaches aim to reduce the GHG footprint of hospital foodservices.

Understanding the perspectives of staff working in hospital foodservices is crucial in transitioning to sustainable practices that benefits the patients, organization, society, and planet as a whole (16). Healthcare staff have identified food waste from hospital foodservices can’t be completely eliminated, but it is a prioritized waste stream that should be focused on (16, 19, 89). Goonan et al. discovered variability in staff perceptions and habits toward food waste generation challenges the achievement of environmental sustainability in an organization (88). Kitchen staff, such as supervisors and cooks, who are involved more in waste generation and management during daily foodservice operations tend to be more food waste conscious and aware of its social and financial implications, whereas some foodservice associated personnel who are comfortably set in their work routines and practices outside of the kitchen environment might be less mindful of food waste implications (88). Even though there is a desire for change in hospital staff, a lack of support from policy makers was identified, (16) in addition to the local regulations and hospital policies around food safety and quality control that restrict the reuse or repurpose of food surplus and waste generated from healthcare facilities (11, 83, 88). Governments and foodservices authorities can initiate changes by stipulating policies/guidelines or providing economic incentives on sustainable foodservices, (16) which may guide and support hospitals to enforce environmental sustainable initiatives to shape staff attitudes and behaviors toward greener hospital foodservices (87, 88). Effective communication between policy makers, hospital executives and ground level personnel about values of sustainable foodservices and identification of change opportunities is vital to achieve sustainable food systems at a national and global level (16, 88). For instance, conducting training sessions and educational campaigns regarding food waste prevention and management throughout the hospital FSC can raise staff knowledge, awareness and accountability in food waste, and encourage positive behaviors (16, 87, 88, 90). In addition, setting up an environmental advocacy team and feedback system on performance may support the engagement and motivation in making sustainable changes at all levels of staff (16, 88). Forming coalition with non-governmental organizations targeting at reducing the healthcare environmental footprint (e.g., Health Care Without Harm, Practice Greenhealth) can also provide indispensable opportunities for knowledge and resources sharing to support sustainable practices in healthcare facilities (16, 87, 90).

### 4.1 Limitations

This study analyzes data from a previous systematic review, which included both peer-reviewed journal articles and gray literature on food and food-related waste management practices in global hospital foodservices serving patients, staff and visitors (11). The majority of the data in this review was from gray literature where the quality of research methods and reporting of findings may be lower than peer reviewed research, as criteria for reporting or quality assessment are lacking. Gray literature sources (e.g., case studies) remain a useful method to disseminate lessons learned and evaluation outcomes about food waste management strategies to help other institutions in decision making processes (11, 18).

Due to the variance in sample sizes under different waste management scenarios, the research team accommodated this by calculating the percentage change in GHG footprint within and between each scenario for more meaningful comparisons. However, the limited samples in some cases made it impossible to conduct a full comparison between all eight food waste management scenarios. Additionally, selecting samples based on reporting of food waste in weight with a specified time-period might introduce sampling bias and undermine the sample representativeness for generalizability. In addition, several assumptions were made in the methodology in this study, including assuming sending food waste to landfill was the current situation if the original food waste destination was not mentioned, and using an average of food waste quantity for cases accounted for multiple facilities. This may have misestimated the actual waste quantity in a hospital diverted in the included hospitals. The ReFED tool was used for calculating the GHG footprint and it contains its own assumptions, thus other GHG impact tools may estimate GHG emissions differently.

There is no investigation on GHG emissions from food-related waste (e.g., packaging materials, disposable crockery), coffee grounds and oil waste in this study. This is one of the limitations of the ReFED tool which focuses solely on food waste and might underestimate the potential waste diversion circumstances in facilities. Food-related waste constitutes a considerable amount of waste in hospital foodservices (24) and have significant impact on the environment (91). Future studies should consider GHG footprint from this waste stream and investigate hospital foodservices waste on other environmental and social impacts (e.g., water use, energy consumption, land demand, meal recovery). Cost-benefit or cost-effective analysis of waste diversion strategies in hospital foodservices will also be helpful for healthcare organizations to determine feasible evidence-based solutions. Moreover, this study contains primarily hospitals in developed countries (especially the US), and there is large capacity for future investigation on hospital foodservice food waste diversion in other countries and the impacts on sustainability.

## 5 Conclusion

The escalating concerns on global food waste and its implications to environmental sustainability has created research interests in hospital foodservices locally and globally, due to their considerable potential for food waste generation and associated GHG emissions. This study demonstrates various food waste diversion strategies have different capabilities in handling the amounts of food waste, yet each strategy displays a reduction in GHG emissions when replaced by a higher prioritized strategy in the food recovery hierarchy (23). There are site-specific factors to consider for the most feasible approach, such as available resources or funding for sustainable waste management practices, collaboration opportunities with donation programs, and availability of composting or industrial facilities (18, 87). Based on the current study and existing literature, food waste prevention in the first place is the most beneficial and sustainable approach. For food waste that is unavoidable, the best approach to manage this is via surplus food donations which demonstrate the greatest reduction in GHG emissions, followed by other waste diversion strategies, including composting and industrial uses. Tackling food waste in hospital foodservices using strategies from the food recovery hierarchy (23) framework is highly encouraged due to their great potential to reduce GHG footprint in a hospital foodservice setting, and subsequently lead to environmental and socioeconomic sustainability in healthcare facilities.

## Data Availability

Publicly available datasets were analyzed in this study. This data can be found here: Cook et al. (11).

## Footnotes

1
https://insights-engine.refed.org/impact-calculator?emissionsEquivalence=milesDriven&impactMetricTab=emissions&inputs=%7B%22sector%22%3Anull%2C%22type%22%3Anull%2C%22unit%22%3A%22tons%22%2C%22destinations%22%3A%5B%5D%7D&isFormExpanded=false&methaneEquivalence=milesDriven&year=100

## References

[1] Mikhaylov A Moiseev N Aleshin K Burkhardt T. Global climate change and greenhouse effect. *Entrepreneursh Sustain Issues.* (2020) 7:2897. 10.9770/jesi.2020.7.4(21)

[2] Yoro K Daramola M. Chapter 1 - CO2 emission sources, greenhouse gases, and the global warming effect. In: Rahimpour M Farsi M Makarem M editors. *Advances in Carbon Capture.* Sawston: Woodhead Publishing (2020). p. 3–28. 10.1016/B978-0-12-819657-1.00001-3

[3] Jackson R Le Quéré C Andrew R Canadell J Peters G Roy J Warning signs for stabilizing global CO2 emissions. *Environ Res Lett.* (2017) 12:110202. 10.1088/1748-9326/aa9662

[4] Carino S Porter J Malekpour S Collins J. Environmental sustainability of hospital foodservices across the food supply chain: A systematic review. *J Acad Nutr Diet.* (2020) 120:825–73. 10.1016/j.jand.2020.01.001 32093919

[5] United States Environmental Protection Agency. *Basic Information about Landfill Gas.* (2023). Available online at: https://www.epa.gov/lmop/basic-information-about-landfill-gas#methane (accessed May 7, 2023).

[6] Thiel C Park S Musicus A Agins J Gan J Held J Waste generation and carbon emissions of a hospital kitchen in the US: Potential for waste diversion and carbon reductions. *PLoS One.* (2021) 16:e0247616. 10.1371/journal.pone.0247616 33730046 PMC7968671

[7] United States Environmental Protection Agency. *Global Methane Initiative: Importance of Methane.* (2022). Available online at: https://www.epa.gov/gmi/importance-methane (accessed May 4, 2023).

[8] Papargyropoulou E Lozano K Steinberger J Wright N Ujang Z. The food waste hierarchy as a framework for the management of food surplus and food waste. *J Clean Prod.* (2014) 76:106–15. 10.1016/j.jclepro.2014.04.020

[9] Food and Agriculture Organization of the United Nations (FAO). *Food Wastage Footprint Impacts on Natural Resources Summary Report.* (2013). Available online at: https://www.fao.org/docrep/018/i3347e/i3347e.pdf (accessed April 16, 2023).

[10] Food and Agriculture Organization of the United Nations (FAO). *Global food Losses and Food Waste Extent, Causes and Prevention.* (2011). Available online at: http://www.fao.org/3/a-i2697e.pdf (accessed April 16, 2022).

[11] Cook N Goodwin D Porter J Collins J. Food and food-related waste management strategies in hospital food services: A systematic review. *Nutr Dietetics.* (2022) 80:116–42. 10.1111/1747-0080.12768 36168297

[12] Food and Agriculture Organization of the United Nations (FAO). *Food Wastage Footprint & Climate Change.* (2023). Available online at: http://www.fao.org/3/a-bb144e.pdf (accessed April 16, 2023).

[13] United Nations Sustainable Development Goals. *Sustainable Development Goal 12: Ensure Sustainable Consumption and Production Patterns.* (2023). Available online at: https://sustainabledevelopment.un.org/sdg12 (accessed April 19, 2023).

[14] Defra. *Government Review of Waste Policy in England 2011: Department for Environment, Food and Rural Affairs.* (2011). Available online at: https://www.gov.uk/government/publications/government-review-of-waste-policy-in-england-2011 (accessed April 19, 2023).

[15] Australian Government. *Food Waste Strategy—HALVING Australia’s Food Waste by 2030.* (2017). Available online at: https://www.dcceew.gov.au/sites/default/files/documents/national-food-waste-strategy.pdf (accessed April 19, 2023).

[16] Carino S Collins J Malekpour S Porter J. Environmentally sustainable hospital foodservices: Drawing on staff perspectives to guide change. *Sustain Prod Consum.* (2021) 25:152–61. 10.1016/j.spc.2020.08.003

[17] Williams P Walton K. Plate waste in hospitals and strategies for change. *e-SPEN Europ J Clin Nutr Metab.* (2011) 6:e235–41. 10.1016/j.eclnm.2011.09.006

[18] Porter J Collins J. A qualitative study exploring hospital food waste from the patient perspective. *J Nutr Educ Behav.* (2021) 53:410–7. 10.1016/j.jneb.2020.10.008 33526388

[19] Ofei K Holst M Rasmussen H Mikkelsen B. How practice contributes to trolley food waste. A qualitative study among staff involved in serving meals to hospital patients. *Appetite.* (2014) 83:49–56. 10.1016/j.appet.2014.08.001 25108237

[20] Cook N Collins J Goodwin D Porter JA. systematic review of food waste audit methods in hospital foodservices: Development of a consensus pathway food waste audit tool. *J Hum Nutr Dietetics.* (2022) 35:68–80. 10.1111/jhn.12928 34060673

[21] Cook N Collins J Goodwin D Porter J. Factors influencing implementation of food and food-related waste audits in hospital foodservices. *Front Nutr.* (2022) 9:1062619. 10.3389/fnut.2022.1062619 36532534 PMC9753938

[22] Bux C. Conventional and digital technologies for measuring and monitoring food waste in the healthcare foodservice. *J Foodserv Bus Res.* (2024) 27:1–23. 10.1080/15378020.2024.2394730

[23] United States Environmental Protection Agency. *Food Recovery Hierarchy.* (2022). Available online at: https://www.epa.gov/sustainable-management-food/foodrecovery-hierarchy (accessed February 3, 2023).

[24] Collins J Porter J. Quantifying waste and its costs in hospital foodservices. *Nutr Diet.* (2023) 80:192–200. 10.1111/1747-0080.12796 36690908

[25] Eriksson M Strid I Hansson P. Carbon footprint of food waste management options in the waste hierarchy–a Swedish case study. *J Clean Prod.* (2015) 93:115–25. 10.1016/j.jclepro.2015.01.026

[26] United States Environmental Protection Agency. *Food: Material-Specific Data [Internet] United States2020.* (2023). Available online at: https://www.epa.gov/facts-and-figures-about-materials-waste-and-recycling/food-material-specific-data (accessed April 24, 2023).

[27] United States Environmental Protection Agency. *Understanding Global Warming Potentials.* (2025). Available online at: https://www.epa.gov/ghgemissions/understanding-global-warming-potentials (accessed March 4, 2025).

[28] United States Environmental Protection Agency. *Sources of Greenhouse Gas Emissions.* (2025). Available online at: https://www.epa.gov/ghgemissions/sources-greenhouse-gas-emissions (accessed March 4, 2025).

[29] ReFED. *Our Work Data & Insights New York.* (2023). Available online at: https://refed.org/our-work/data-and-insights (accessed February 3, 2023).

[30] Quantis. *Greenhouse Gas Emissions of Food Waste: Methodology United States.* (2024). Available online at: https://refed.org/downloads/quantis-greenhouse-gas-emissions-of-food-waste-methodology/ (accessed March 4, 2025).

[31] ReFED. *Insights Engine Impact Calculator Methodology New York.* (2024). Available online at: https://docs.refed.org/methodologies/impact_calculator/methodology_pdfs.html#impact-calculator-methodology-pdfs (accessed March 4, 2025).

[32] ReFED. *Insights Engine Solutions Database Methodology.* (2020). Available online at: https://insights.refed.org/methodology?section=impact-calculator (accessed February 3, 2023).

[33] ReFED. *Insights Engine Glossary.* (2023). Available online at: https://docs.refed.org/glossary.html (accessed February 3, 2023).

[34] Biocycle. *Composting Roundup.* (2015). Available online at: https://www.biocycle.net/composting-roundup-56/ (accessed February 4, 2023).

[35] Ohio State University. *Closing in ON zero-Waste Goals with Sustainable Food Prep 2019.* (2023). Available online at: https://si.osu.edu/closing-zero-waste-goals-sustainable-food-prep (accessed February 4, 2023).

[36] Ramsay Health Care Limited. *Impact Report FY2019.* (2019). Available online at: https://www.ramsayhealth.com/en/investors/results-and-reports/ (accessed February 5, 2023).

[37] Global Green and Healthy Hospitals. *Reducing Hunger and Food Waste in our Community Melbourne Health, Australia.* (2020). Available online at: https://www.greenhospitals.net/wp-content/uploads/2020/01/GGHH-Case-Study-Reducing-hunger-and-food-waste-in-our-community-Melbourne-Health.pdf (accessed February 5, 2023).

[38] Smith M. *How Unused Hospital Food is Feeding the Hungry in NorCal.* (2020). Available online at: https://vitals.sutterhealth.org/hungry-people-fed-through-food-waste-reduction-pilot/ (accessed February 4, 2023).

[39] Epworth Health. *Turning Waste to Green Energy.* (2020). Available online at: https://www.epworth.org.au/blog/2020/turning-waste-to-green-energy (accessed February 4, 2023).

[40] Neale H. *Environment: a Useful Solution in Waste.* (2019). Available online at: https://www.braidwoodtimes.com.au/story/6025186/a-useful-solution-in-waste/ (accessed February 4, 2023).

[41] Greenwalt M. *A Digestion System Helped one Hospital Reach its Zero Waste Goals.* (2016). Available online at: https://www.waste360.com/food-waste/digestion-system-helped-one-hospital-reach-its-zero-waste-goals (accessed February 4, 2023).

[42] Closed Loop. *Closed Loop’s Composting Expertise Helps Barwon Health’s Journey Toward Zero Waste.* (2016). Available online at: https://closedloop.com.au/case-studies-barwon-health/ (accessed February 4, 2023).

[43] Interreg Central Europe Strefowa. *Food Donation from Hospitals to the Territory (Italy).* (2023). Available online at: http://www.reducefoodwaste.eu/food-donation-from-hospitals-to-the-territory.html (accessed February 4, 2023).

[44] Iuguis. *Hollywood Private Hospital.* (2023). Available online at: https://iugis.com/hollywood-private-hospital-case-study (accessed February 4, 2023).

[45] Green Impact. *Three Tips to Reduce Hospital Food Waste.* (2023). Available online at: https://www.greenimpact.com/best-practices-and-tools/three-tips-to-reduce-hospital-food-waste/ (accessed February 4, 2023).

[46] Buzalka M. *Morrison Makes Food Recovery/Donation Commitment.* (2017). Available online at: https://www.food-management.com/healthcare/morrison-makes-food-recoverydonation-commitment (accessed February 4. 2023).

[47] The University of Vermont Health Network. *Mountain View Cafe.* (2023). Available online at: https://www.cvmc.org/patients-visitors/visitor-information/mountain-view-caf%C3%A9 (accessed February 4, 2023).

[48] Rush. *Rush Surplus Project to Feed More in 2019.* (2019). Available online at: https://www.rushu.rush.edu/news/rush-surplus-project-feed-more-2019#:~:text=An%20initiative%20to%20donate%20excess,patients%20faced%20with%20food%20insecurity (accessed February 4, 2023).

[49] Diariodelweb. *«Biella Solidale»: Hospital Meals for the Most Needy.* (2016). Available online at: https://www.diariodelweb.it/biella/articolo/?nid=20160208_374213 (accessed February 4, 2023).

[50] Kaiser Permanente. *Waste Less Food for a Healthy Planet.* (2019). Available online at: https://about.kaiserpermanente.org/commitments-and-impact/healthy-communities/news# (accessed February 4, 2023).

[51] Galindo Y. *Reducing Food Insecurity Through Food Sustainability.* (2019). Available online at: https://today.ucsd.edu/story/reducing-food-insecurity-through-food-sustainability (accessed February 4, 2023).

[52] Corrigan P. *Waste Not: Hospitals Fight Hunger by Donating Surplus Food to Feed Community Members.* (2019). Available online at: https://www.chausa.org/publications/catholic-health-world/archives/issues/january-15-2019/waste-not-hospitals-fight-hunger-by-donating-surplus-food-to-feed-community-members (accessed February 4, 2023).

[53] Health Care Without Harm. *Workshop Report Sustainable and Healthy Food in Healthcare.* (2018). Available online at: https://noharm-global.org/sites/default/files/documents-files/5818/2018-12-12_CME18_food-workshop-report_FINAL.pdf (accessed February 5, 2023).

[54] McKinney M. *Iowa Medical Center Devises Strategy to Reduce Food Waste.* (2015). Available online at: https://go.gale.com/ps/i.do?p=AONE&u=monash&id=GALE| A421127036&v=2.1&it=r&sid=AONE&asid=1d35677 (accessed February 4, 2023). 30387962

[55] Gerlat A. *Food Waste Recycling Firm Biohitech Expands Digester Business.* (2014). Available online at: https://www.waste360.com/food-waste/food-waste-recycling-firm-biohitech-expands-digester-business (accessed February 4, 2023).

[56] Chumari A. *Food Waste Not Wasted.* (2018). Available online at: https://www.singhealth.com.sg/news/joy-at-work/18-january-2018 (accessed February 4, 2023).

[57] Parkland Health & Hospital System. *Parkland Food ‘Digester’ Takes Bite out of Landfill.* (2014). Available online at: https://www.parklandhealth.org/news-and-updates/parkland-food-digester-takes-bite-out-of-landfill-1 (accessed February 4, 2023).

[58] Power Knot. *Toronto Hospital uses Biodigester to Turn Leftover Food Scraps into Drain-Safe Grey Water.* (2018). Available online at: https://powerknot.com/2018/09/08/toronto-hospital-biodigests-leftovers-turns-food-scraps-into-drain-saft-grey-water/ (accessed February 4, 2023).

[59] England Sustainable. *Development Unit (National Health Service). Case Study Food Waste Stockport.* (2023). Available online at: https://www.sduhealth.org.uk/documents/case_study/Food%20Waste.pdf (accessed February 4, 2023).

[60] Practice Greenhealth. *Food Digester.* (2023). Available online at: https://practicegreenhealth.org/sites/default/files/upload-files/awards/resources/leadership_example_of_roi_or_green_revolving_fund_northshore_university_health_system_2016.pdf (accessed February 5, 2023).

[61] Hensley B. *Sustainabilty What Can I do? Presentation.* (2021). Available online at: https://www.ihhc.org.au/conference/2021-conference/program/session-information/ (accessed February 4, 2023).

[62] Waddington K. *Taking a Bite out of Organic Waste.* (2013). Available online at: https://greenhealthcare.ca/wp-content/uploads/2017/07/CCGHC-Organic-Waste-Case-Study-June17-2013-FINAL.pdf (accessed February 5, 2023).

[63] Metropolitan Waste and Resource Recovery Group. *Recycling Hospital Food Waste Department of Health and Human Services.* (2017). Available online at: https://www.mwrrg.vic.gov.au/assets/resource-files/Organics-Dept-of-Health-recycling-food-waste-new.pdf (accessed February 5, 2023).

[64] Green Eco Technologies. *Case Study: WasteMaster Solves Hospital Problems Caused by Food Waste.* (2018). Available online at: https://www.greenecotec.com/successstories/kettering-general-hospital (accessed February 4, 2023).

[65] do Nascimento KS da Silva S Santos E. Geraç~ao de energia elétrica e viabilidade técnico-econômica de um biodigestor no setor hospitalar. *Pubvet.* (2017) 11:1188–297. 10.22256/PUBVET.V11N12.1263-1273

[66] National Health Service. *Sustainability Impact Report 2019.* (2019). Available online at: https://www.nhssustainabilityday.co.uk/wp-content/uploads/2019/07/SD-Impact-report-2019.pdf (accessed February 4, 2023).

[67] Massachusettes General Hospital. *Mass general composting program turns food waste into energy.* (2021). Available online at: https://www.massgeneral.org/news/hotline/htl033121/composting (accessed February 4, 2023).

[68] Clugston H. *Revealed: The NHS Hospitals Throwing Away Thousands of Tonnes of Food Waste Every Year.* (2021). Available online at: https://www.nationalworld.com/news/uk-news/revealed-the-nhs-hospitals-throwing-away-thousands-of-tonnes-of-food-waste-every-year-3211575 (accessed February 4, 2023).

[69] Galvan AH George D. Repurposing waste streams: Lessons on integrating hospital food waste into a community garden. *J Community Health.* (2018) 43:944–6. 10.1007/s10900-018-0509-x 29623502

[70] Jamieson CW Ozores-Hampton M Nutter J Thavarajah B. *Collection and Diversion of Food Residuals in Southwest Florida.* (2004). Available online at: https://www.biocycle.net/collection-and-diversion-of-food-residuals-in-southwest-florida/ (accessed February 4, 2023).

[71] Emerson D. *Federal agencies Get with the Food Recycling Program.* (2013). Available online at: https://www.biocycle.net/federal-agencies-get-with-the-food-recycling-program/ (accessed February 4, 2023).

[72] Lehman D. Waste away. Composting programs take hold at pioneering health care facilities. *Health Facil Manage.* (2003) 16:33–6. 14710688

[73] Wrobel S. *Hospitals Turn Food Waste into Compost.* (2010). Available online at: https://www.emory.edu/EMORY_REPORT/stories/2010/04/19/hospital_compost.html (accessed February 4, 2023).

[74] Carvalho A. *Hospital Chain on Board with Food Scraps Diversion.* (2012). Available online at: https://www.biocycle.net/hospital-chain-on-board-with-food-scraps-diversion/ (accessed February 4, 2023).

[75] Zero Waste S. *Leadership in Hospital Waste Management.* (2023). Available online at: https://www.greenindustries.sa.gov.au/_literature_165590/Royal_Adelaide_Hospital_and_Zero_Waste_SA (accessed February 5, 2023).

[76] Tasmania R. *Waste NoT Awards 2020 Winners.* (2020). Available online at: https://rethinkwaste.com.au/waste-not-awards-2020-winners/ (accessed February 4, 2023).

[77] Soares D Chagas M. *Use of the Composting Technique in the Nutrition and Service Dietetics at Hospital Municipal Pimentas Bonsucesso.* (2023). Available online at: http://www.hospitaissaudaveis.org/arquivos/COMPOSTAGEM%202.pdf (accessed February 6, 2023).

[78] Premier. *Bon Secours St. Francis Health System Recycling, Waste Reductions Efforts Yield Rapid Success.* (2023). Available online at: http://www.premiersafetyinstitute.org/wp-content/uploads/StFranci–BonSecours-WasteRecycling.pdf (accessed February 4, 2023).

[79] Purdy T. *Designing and Implementing a Hospital Environmental Management Framework.* Windsor, ON: University of Windsor (2013).

[80] Waste and Resources Action Programme. *Case study: Somerset partnership NHS Foundation Trust Food Waste Collection.* (2014). Available online at: https://www.wrap.org.uk/sites/files/wrap/Somerset_Trust_NHS_case_study.pdf (accessed February 6, 2023).

[81] Waste and Resources Action Programme. *Case Study: NHS Ayrshire and Arran food waste Collection.* (2014). https://www.wrap.org.uk/sites/files/wrap/Ayrshire_Arran_NHS_case_study.pdf (accessed February 6, 2023).

[82] Samantha Lomuscio. *Research Data Services + Sciences - Getting Started with the Kruskal-Wallis Test.* (2021). Available online at: https://data.library.virginia.edu/getting-started-with-the-kruskal-wallis-test/#:~:text=Sample%20size%20%E2%80%93%20each%20group%20must,size%20of%205%20or%20more (accessed April 28, 2023).

[83] Muth M Birney C Cuéllar A Finn S Freeman M Galloway J A systems approach to assessing environmental and economic effects of food loss and waste interventions in the United States. *Sci Total Environ.* (2019) 685:1240–54. 10.1016/j.scitotenv.2019.06.230 31390713 PMC7343133

[84] Van Der Werf P Seabrook J Gilliland J. The quantity of food waste in the garbage stream of southern Ontario, Canada households. *PLoS One.* (2018) 13:e0198470. 10.1371/journal.pone.0198470 29897964 PMC5999097

[85] Cornell Law School Legal Information Institute. *Bill Emerson Good Samaritan Food Donation Act.* (2023). Available online at: https://www.law.cornell.edu/uscode/text/42/1791 (accessed May 6, 2023).

[86] United States Environmental Protection Agency. *Wasted Food Measurement Methodology Scoping Memo.* (2020). Available online at: https://www.epa.gov/sites/production/files/2020-06/documents/food_measurement_methodology_scoping_memo-6-18-20.pdf (accessed April 30, 2023).

[87] Saber DA Azizi R Drever S Sanford D Nadeau H. Hospital food waste: Reducing waste and cost to our health care system and environment. *Online J Issues Nurs.* (2022) 27:1–11. 10.3912/OJIN.Vol27No01PPT33

[88] Goonan S Mirosa M Spence H. Getting a taste for food waste: A mixed methods ethnographic study into hospital food waste before patient consumption conducted at three New Zealand foodservice facilities. *J Acad Nutr Diet.* (2014) 114:63–71. 10.1016/j.jand.2013.09.022 24231365

[89] Victorian Department of Health - Department of Health and Human Services. *Waste Education in Health Care: Summary Report.* (2018). Available online at: https://www.health.vic.gov.au/publications/waste-education-in-health-care-summary-report (accessed May 2, 2023).

[90] Wilson ED Garcia AC. Environmentally friendly health care food services: A survey of beliefs, behaviours, and attitudes. *Can J Diet Pract Res.* (2011) 72:117–22. 10.3148/72.3.2011.117 21896245

[91] Bala A Laso J Abejón R Margallo M Fullana-i-Palmer P Aldaco R. Environmental assessment of the food packaging waste management system in Spain: Understanding the present to improve the future. *Sci Total Environ.* (2020) 702:134603. 10.1016/j.scitotenv.2019.134603 31726337

