# Supplementary material for: Food waste management practices in hospital foodservices and their associated greenhouse gas emissions: potential for increased environmental sustainability
Source: Front Nutr. 2025 May 13;12:1541657. doi: 10.3389/fnut.2025.1541657 (PMC12106006; doi:10.3389/fnut.2025.1541657)
Supplement: Supplementary file 1 [file Table_1.docx]

***Supplementary Material***

**Table 1. Case studies characteristics, GHG emissions and net change using different food waste management strategies in hospital foodservices in a one-year period.**

| Citation (Author, year) | Location of facility | Number of facilities | Setting | Food waste source | Current strategy | Alternative strategy | Food waste amount (kg) | GHG (MTCO_2_e) Current strategy | GHG (MTCO_2_e) Alternative strategy | GHG (MTCO_2_e) Net change^#^  (% change) |
| --- | --- | --- | --- | --- | --- | --- | --- | --- | --- | --- |
| **Composting to Surplus Food Donations** | | | | | | | | | | |
| Green Impact(55) | USA | 1 | Medical center | Patient food service | Composting | Donation | 10886.4 | 53.26 | 4.65 | - 48.61  (- 91.27%) |
| Buzalka, 2017(56) | USA | 1 | Hospital | Patient food service | Composting | Donation | 1180.4 | 5.78 | 0.5 | - 5.28  (- 91.35%) |
| **Landfill to Surplus Food Donations** | | | | | | | | | | |
| The University of Vermont Health Network(57) | USA | 1 | Medical center | Cafeteria | Assumed landfill | Donation | 1092 | 5.85 | 0.47 | - 5.38  (- 91.97%) |
| Rush, 2019(58) | USA | 1 | Hospital | Cafeteria | Assumed landfill | Donation | 1632 | 8.74 | 0.7 | - 8.04  (- 91.99%) |
| Diariodelweb, 2016(59) | Italy | 1 | Hospital | Canteen | Assumed landfill | Donation | 3628.74 | 19.44 | 1.55 | - 17.89  (- 92.03%) |
| Kaiser Permanente, 2019(60) | USA | 1 | Medical center | Patient food service | Assumed landfill | Donation | 6350 | 34.02 | 2.71 | - 31.31  (- 92.03%) |
| Interreg Central Europe Strefowa(43) | Italy | 1 | Hospital | Canteen | Landfill | Donation | 2568 | 13.76 | 1.1 | - 12.66  (- 92.01%) |
| Galindo, 2019(61) | USA | 2 | Medical center | Patient food service | Landfill | Donation | 2000* | 10.72* | 0.86* | - 9.86*  (- 91.98%) |
| Global Green and Healthy Hospitals, 2020(37) | Australia | 1 | Hospital, CPK | Patient food service | Landfill | Donation | 8278.06 | 44.36 | 3.54 | - 40.82  (- 92.02%) |
| Corrigan, 2019(62) | USA | 1 | Hospital | Patient food service, cafeteria/canteen/restaurant | Assumed landfill | Donation | 2832 | 15.17 | 1.21 | - 13.96  (- 92.02%) |
| Corrigan, 2019(62) | USA | 2 | Medical center | Patient food service, cafeteria/canteen/restaurant | Assumed landfill | Donation | 1177.5* | 6.31* | 0.51* | - 5.81*  (- 92.08%) |
| Ramsay Health Care Limited, 2019(36) | Australia | 1 | Hospital | Patient food service | Assumed landfill | Donation | 624 | 3.34 | 0.27 | - 3.07  (- 91.92%) |
| Health Care Without Harm, 2018(63) | Spain | 1 | Hospital | Patient food service, cafeteria/canteen/restaurant | Assumed landfill | Donation | 2340 | 12.54 | 1 | - 11.54  (- 92.03%) |
| McKinney, 2015(64) | USA | 1 | Medical center | Patient food service | Landfill | Donation | 2041 | 10.94 | 0.87 | - 10.07  (- 92.05%) |
| Ohio State University, 2019(35) | USA | 1 | Medical center | Patient food service | Assumed landfill | Donation | 18144 | 97.22 | 7.75 | - 89.47  (- 92.03%) |
| Ramsay Health Care Limited, 2019(36) | Australia | 1 | Hospital | Patient food service | Landfill | Donation | 4305 | 23.07 | 1.84 | - 21.23  (- 92.02%) |
| Smith, 2020(38) | USA | 16 | Health service | Patient food service | Landfill | Donation | 1899.38* | 10.18* | 0.81* | - 9.37*  (- 92.04%) |
| **Landfill to Animal Feed** | | | | | | | | | | |
| Neale, 2019(40) | Australia | 1 | Hospital | Patient food service | Landfill | Animal feed | 2540.12 | 13.61 | 12.81 | - 0.8  (- 5.88%) |
| **Landfill to Industrial Uses** | | | | | | | | | | |
| Greenwalt, 2016(41) | USA | 1 | Hospital | Patient food service, cafeteria/canteen/restaurant | Landfill | Industrial uses | 56699 | 303.81 | 280.26 | - 23.55  (- 7.75%) |
| Gerlat, 2014(65) | USA | 1 | Medical center | Patient food service | Assumed landfill | Industrial uses | 119748.36 | 641.64 | 591.91 | - 49.73  (- 7.75%) |
| Chumari, 2018(66) | Singapore | 1 | Hospital | Patient food service | Assumed landfill | Industrial uses | 264625 | 1417.93 | 1308.02 | - 109.91  (- 7.75%) |
| Parkland Health and Hospital System, 2004(67) | USA | 1 | Health service | Patient food service | Landfill | Industrial uses | 326587 | 1749.94 | 1614.3 | - 135.64  (- 7.75%) |
| Power knot, 2018(68) | Canada | 1 | Hospital | Patient food service | Landfill | Industrial uses | 40150 | 215.13 | 198.46 | - 16.67  (- 7.75%) |
| Iugis(44) | Australia | 1 | Hospital | Patient food service | Assumed landfill | Industrial uses | 90718.5 | 486.09 | 448.41 | - 37.68  (- 7.75%) |
| NHS England Sustainable development Unit(69) | UK | 1 | Hospital | Patient food service | Assumed landfill | Industrial uses | 62400 | 334.36 | 308.44 | - 25.92  (- 7.75%) |
| Practice Greenhealth(70) | USA | 1 | Hospital | Patient food service | Landfill | Industrial uses | 136078 | 729.14 | 672.62 | - 56.52  (- 7.75%) |
| Hensley, 2021(71) | Australia | 1 | Hospital | Patient food service | Landfill | Industrial uses | 48000 | 257.2 | 237.26 | - 19.94  (- 7.75%) |
| Global Green and Healthy Hospitals, 2020(37) | Australia | 1 | Hospital | Patient food service | Assumed landfill | Industrial uses | 157850.16 | 845.8 | 780.24 | - 65.56  (- 7.75%) |
| Waddington, 2013(72) | Canada | 1 | Hospital | Patient food service | Landfill | Industrial uses | 27215.5 | 145.83 | 134.52 | - 11.31  (- 7.76%) |
| Epworth Health, 2020(39) | Australia | 1 | Hospital | Patient food service | Landfill | Industrial uses | 18143.7 | 97.22 | 89.68 | - 7.54  (- 7.76%) |
| Metropolitan Waste and Resource Recovery Group, 2017(73) | Australia | 1 | Hospital | Patient food service | Landfill | Industrial uses | 281227.35 | 1506.89 | 1390.09 | - 116.8  (- 7.75%) |
| Green Eco Technologies, 2018(74) | UK | 1 | Hospital | Patient food service | Assumed landfill | Industrial uses | 45359.2 | 243.05 | 224.21 | - 18.84  (- 7.75%) |
| do Nascimento et al. 2017(75) | Brazil | 1 | Hospital | Patient food service | Landfill | Industrial uses | 20916 | 112.07 | 103.39 | - 8.68  (- 7.75%) |
| **Landfill to Anaerobic Digestion** | | | | | | | | | | |
| NHS England (National Health Service), 2019(76) | UK | 1 | Hospital | Patient food service | Landfill | Anaerobic digestion | 130634.4 | 699.97 | 653.85 | - 46.12  (- 6.59%) |
| Massachusetts General Hospital, 2021(77) | USA | 1 | Hospital | Cafeteria | Assumed landfill | Anaerobic digestion | 474458 | 2542.27 | 2374.75 | - 167.52  (- 6.59%) |
| Clugston, 2021(78) | UK | 207 | Health service | Patient food service, cafeteria/canteen/restaurant | Assumed landfill | Anaerobic digestion | 61421.23* | 329.11* | 307.42* | - 21.69*  (- 6.59%) |
| **Landfill to Composting** | | | | | | | | | | |
| Galvan et al. 2018(79) | USA | 1 | Hospital | Patient food service | Assumed landfill | Composting | 2358 | 12.63 | 11.54 | - 1.09  (- 8.63%) |
| Jamieson et al. 2004(80) | USA | 1 | Hospital | Cafeteria | Landfill | Composting | 11321.664 | 60.66 | 55.39 | - 5.27  (- 8.69%) |
| Emerson, 2013(81) | USA | 1 | Medical center | Patient food service | Landfill | Composting | 32730.6 | 175.38 | 160.14 | - 15.24  (- 8.69%) |
| Lehman, 2003(82) | USA | 1 | Medical center | Patient food service, cafeteria/canteen/restaurant | Landfill | Composting | 66065 | 353.99 | 323.23 | - 30.76  (- 8.69%) |
| Wrobel, 2010(83) | USA | 1 | Hospital | Patient food service | Landfill | Composting | 43548 | 233.34 | 213.06 | - 20.28  (- 8.69%) |
| Ramsay Health Care Limited, 2019(36) | Australia | 1 | Hospital | Patient food service, cafeteria/canteen/restaurant | Landfill | Composting | 32730.6 | 175.38 | 160.14 | - 15.24  (- 8.69%) |
| Waddington, 2013(72) | Canada | 1 | Hospital | Patient food service | Landfill | Composting | 15966.46 | 85.55 | 78.12 | - 7.43  (- 8.68%) |
| Carvalho, 2012(84) | USA | 2 | Hospital | Patient food service, cafeteria/canteen/restaurant | Assumed landfill | Composting | 82553.9* | 442.35* | 403.9* | - 38.45*  (- 8.69%) |
| Zero Waste SA(85) | Australia | 1 | Hospital | Patient food service | Landfill | Composting | 197766 | 1059.68 | 967.58 | - 92.1  (- 8.69%) |
| Rethink Waste Tasmania, 2020(86) | Australia | 1 | Hospital | Patient food service | Landfill | Composting | 91000 | 487.6 | 445.22 | - 42.38  (- 8.69%) |
| Biocycle, 2015(34) | USA | 1 | Health service | Canteen | Assumed landfill | Composting | 10886.2 | 58.33 | 53.26 | - 5.07  (- 8.69%) |
| Closed Loop, 2016(42) | Australia | 1 | Health service, CPK | Patient food service | Landfill | Composting | 115212 | 617.34 | 563.68 | - 53.66  (- 8.69%) |
| Soares & Chagas(87) | Brazil | 1 | Hospital | Patient food service | Assumed landfill | Composting | 10886.22 | 58.33 | 53.26 | - 5.07  (- 8.69%) |
| Waddington, 2013(72) | Canada | 1 | Hospital | Patient food service | Landfill | Composting | 4336 | 23.23 | 21.21 | - 2.02  (- 8.70%) |
| Premier(88) | USA | 2 | Health service | Patient food service | Assumed landfill | Composting | 136* | 0.73* | 0.67* | - 0.06*  (- 8.22%) |
| Ohio State University, 2019(35) | USA | 1 | Medical center | Patient food service | Landfill | Composting | 40823 | 218.74 | 199.73 | - 19.01  (- 8.69%) |
| Purdy, 2013(89) | Canada | 1 | Hospital | Patient food service, cafeteria/canteen/restaurant | Landfill | Composting | 75296.3 | 403.46 | 368.39 | - 35.07  (- 8.69%) |
| **Sewer to Anaerobic Digestion** | | | | | | | | | | |
| WRAP, 2014(90) | UK | 1 | Hospital | Patient food service, cafeteria/canteen/restaurant | Sewer | Anaerobic digestion | 9295.15 | 52.67 | 46.52 | - 6.15  (- 11.68%) |
| **Sewer to Composting** | | | | | | | | | | |
| WRAP, 2014(91) | UK | 2 | Hospital | Patient food service, cafeteria/canteen/restaurant | Sewer | Composting | 34926.6* | 197.90* | 170.88* | - 27.02*  (- 13.65%) |

*average values were displayed to account for multiple sites (n=7)

^#^expressed as net amount of GHG savings from current strategy (i.e., net change in GHG footprint = alternative strategy GHG – current strategy GHG)
